# Cost-effectiveness of hepatitis C treatment using generic direct-acting antivirals available in India

**DOI:** 10.1371/journal.pone.0176503

**Published:** 2017-05-17

**Authors:** Rakesh Aggarwal, Qiushi Chen, Amit Goel, Nicole Seguy, Razia Pendse, Turgay Ayer, Jagpreet Chhatwal

**Affiliations:** 1 Department of Gastroenterology, Sanjay Gandhi Postgraduate Institute of Medical Sciences, Lucknow, India; 2 Massachusetts General Hospital Institute for Technology Assessment, Boston, Massachusetts, United States of America; 3 Harvard Medical School, Boston, Massachusetts, United States of America; 4 World Health Organization India Country Office, New Delhi, India; 5 World Health Organization Regional Office for the South-East Asia, Communicable Diseases Department, New Delhi, India; 6 H. Milton Stewart School of Industrial and Systems Engineering, Georgia Institute of Technology, Atlanta, Georgia, United States of America; University of North Carolina at Chapel Hill School of Dentistry, UNITED STATES

## Abstract

**Background & aims:**

Availability of directly-acting antivirals (DAAs) has changed the treatment landscape of hepatitis C virus (HCV) infection. The high price of DAAs has restricted their use in several countries. However, in some countries such as India, generic DAAs are available at much cheaper price. This study examined whether generic DAAs could be cost-saving and how long it would take for the treatment to become cost-saving/effective.

**Methods:**

A previously-validated, mathematical model was adapted to the HCV-infected population in India to compare the outcomes of no treatment versus treatment with DAAs. Model parameters were estimated from published studies. Cost-effectiveness of HCV treatment using available DAAs was calculated, using a payer’s perspective. We estimated quality-adjusted life years (QALYs), disability-adjusted life years (DALYs), total costs, and incremental cost-effectiveness ratio of DAAs versus no treatment. One-way and probabilistic sensitivity analyses were conducted.

**Results:**

Compared with no treatment, the use of generic DAAs in Indian HCV patients would increase the life expectancy by 8.02 years, increase QALYs by 3.89, avert 19.07 DALYs, and reduce the lifetime healthcare costs by $1,309 per-person treated. Treatment became cost-effective within 2 years, and cost-saving within 10 years of its initiation overall and within 5 years in persons with cirrhosis. Treating 10,000 HCV-infected persons could prevent 3400–3850 decompensated cirrhosis, 1800–2500 HCC, and 4000–4550 liver-related deaths. The results were sensitive to the costs of DAAs, pre- and post-treatment diagnostic tests and management of cirrhosis, and quality of life after sustained virologic response.

**Conclusions:**

Treatment with generic DAAs available in India will improve patient outcomes, provide a good value for money within 2 years, and be ultimately cost-saving. Therefore, in this and similar settings, HCV treatment should be a priority from a public health as well an economic perspective.

## Introduction

Hepatitis C virus (HCV) infects more than 70 million people worldwide and between 6–11 million in India [1]. Chronic HCV infection is the leading cause for cirrhosis, hepatocellular carcinoma (HCC) and liver-related deaths worldwide. Untreated HCV infection also leads to substantial economic burden [2]. In India, HCV infection was estimated to be responsible for 59,000 deaths in the year 2015 [3].

New directly-acting antivirals (DAAs) for HCV treatment are highly effective, with DAA-based regimens providing rates of sustained virological response (SVR) exceeding 95%. These regimens are also very safe and convenient, needing administration of oral drugs once or twice daily for 12–24 weeks [4]. Thus, these drugs offer a hope of reducing the burden of HCV. However, these drugs are very costly in several countries, limiting treatment to those with advanced disease. The median nominal price of a 12-week regimen of sofosbuvir across 26 Organisation for Economic Co-operation and Development (OECD) member countries was US$42,017, ranging from US$37,729 in Japan to US$64,680 in the United States [5].

However, in India, three DAAs (sofosbuvir, ledipasvir and daclatasvir) are available from several generic manufacturers at a price as low as $300 [6]. Despite this, treatment rates remain very low in India [1]. This is partially because of limited budget allocated to HCV and lack of data on the cost-effectiveness of HCV treatment. Almost all published cost-effectiveness analyses were conducted in high- and middle-income countries [7–12], where DAAs are costly. While it is expected that the DAAs will be *cost-effective* at low prices prevalent in India, it is not known how long it would take them to become cost-effective, and if DAAs could also be *cost-saving*; i.e. improve life expectancy and reduce costs at the same time. If it were indeed so, this would encourage advocacy by various stakeholders including the patient groups, physicians and public health professionals towards public funding of HCV treatment, and may make it easier for health administrators and political leaders to take such a decision.

We therefore estimated the cost-effectiveness of treatment of HCV-infected persons in India using low-priced DAAs, and evaluated if/when the upfront cost of treatment for HCV infection could result in cost-savings. This question is also of interest for other countries where it may be possible to obtain DAAs at low prices.

## Methods

### Model overview

We have previously developed and validated an individual-level Markov state-transition model, called the *Markov-based Analyses of Treatments for Chronic Hepatitis C* (*MATCH)* [9], using C++, a general-purpose computer programming language. The natural history output from this model has been validated with the results of a multicenter follow-up study of patients with advanced fibrosis, and with previously published cost-effectiveness studies [13–16]. For the current study, we adapted this model to the HCV-infected population in India (*MATCH-India*), following the principles recommended by the World Health Organization (WHO) on economic analyses in the field of viral hepatitis [17].

### Characteristics of base case population

Our base case population included HCV-infected person, aged 35 years, in India. We defined a total of 30 unique patient profiles based on HCV genotype (G1, G3, or G4), and the patient’s sex (male or female) and METAVIR fibrosis score (no fibrosis [F0], portal fibrosis without septa [F1], portal fibrosis with few septa [F2], numerous septa without fibrosis [F3], or cirrhosis [F4]) [18]. The relative frequencies of these profiles in the HCV-infected Indian cohort were based on the available data (Table A in S1 Appendix). Patients with HIV or hepatitis B virus co-infection, and those belonging to special groups at higher risk of HCV reinfection (e.g. hemodialysis, thalassemia, and haemophilia) were excluded. All patients were considered treatment-naïve because the percentage of treatment-experienced patients in India is negligible.

### Treatment regimens and efficacy

The treatment regimens used were based on the DAAs available in India, depending on HCV genotype and fibrosis stage (Table B in S1 Appendix) [19]. Treatment efficacy, adverse events and premature treatment discontinuation rates were modeled on data from recent clinical trials of DAAs in treatment-naïve patients [20–23].

### Natural history of HCV infection

**Fig 1:**
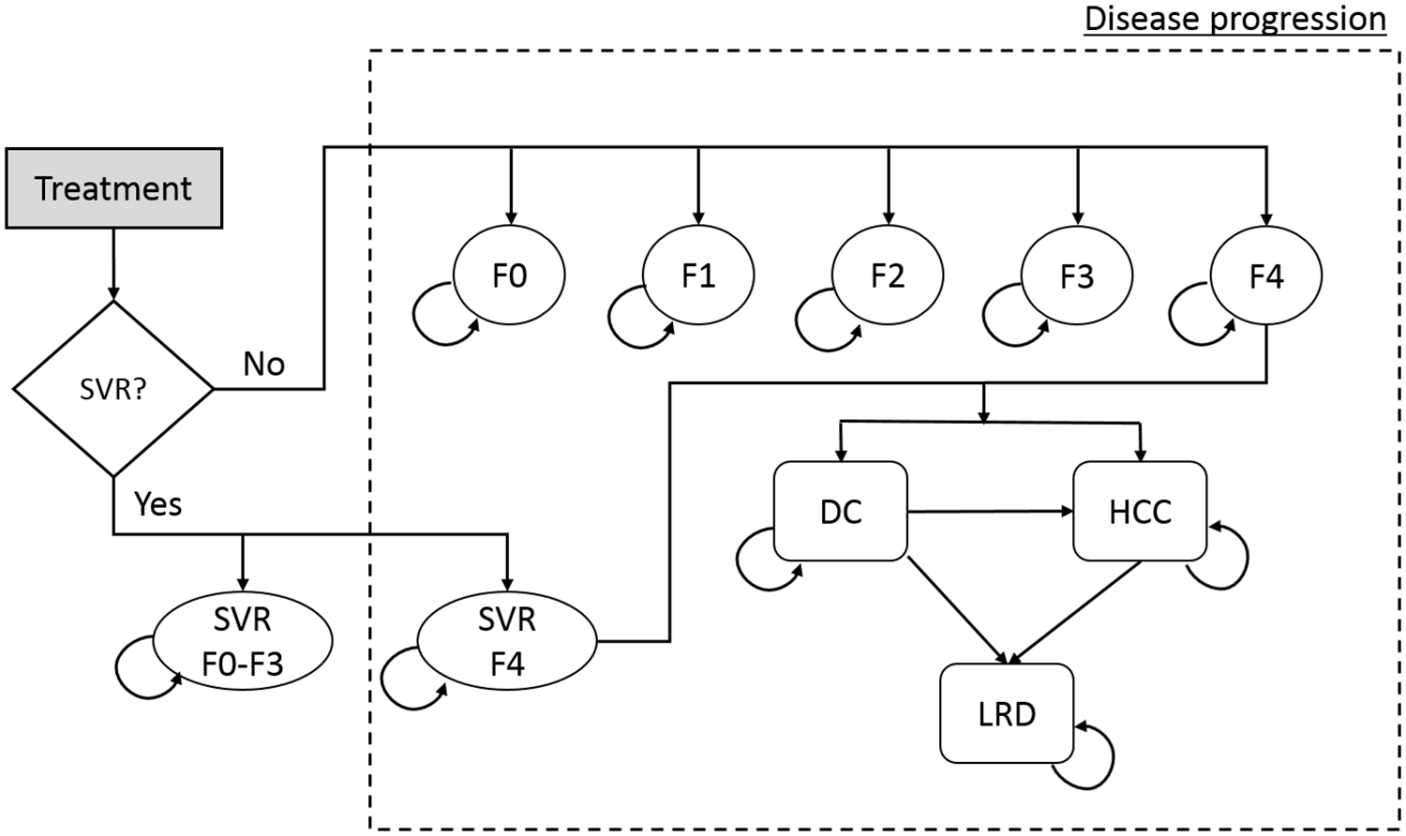
State-transition model schematic of showing the natural history of hepatitis C virus infection. Abbreviations: DC = decompensated cirrhosis; HCC = hepatocellular carcinoma; HCV = hepatitis C virus; LRD = liver-related death; SVR = sustained virologic response. (Adapted from Chhatwal et al.[9])

The natural history of HCV infection and progression were defined as transitions between Markov health states. Each patient started in one of five METAVIR liver fibrosis states (F0–F4) (Fig 1), and could, at the end of each cycle, remain in the same state, die from background mortality, or move into a higher fibrosis state, or to decompensated cirrhosis and/or HCC, or liver-related death. Patients in F0-F3 states who achieved SVR were assumed to be cured and to follow background mortality thereafter; however, those in F4 state who achieved SVR could progress to more advanced states, albeit at a slower rate [24]. Patients who failed to achieve SVR or who discontinued treatment continued to progress over time at the original rate. The cycle length used was one week.

We used data from a previously-published meta-regression analysis to model fibrosis progression rates from F0 to F4 [25], and from published observational studies to model progression from cirrhosis to decompensated cirrhosis and HCC (Table 1) [26, 27]. Patients with decompensated cirrhosis or HCC had a higher liver-related mortality than those in early stages of HCV infection [28]. Because the number of liver transplants performed in India is negligible, our model disregarded the option of liver transplantation.

**Table 1:** Annual transition probabilities, healthcare costs and quality of life weights for different Markov states.

Input	Base case	Values for sensitivity analysis			
		Range	Distribution	Parameter 1^a^	Parameter 2^b^
**Transition *probabilities (annual)***					
F0 to F1 [25]	0.117	0.104–0.130	Beta	274.98	2,075.30
F1 to F2 [25]	0.085	0.075–0.096	Beta	210.06	2,261.18
F2 to F3 [25]	0.120	0.109–0.133	Beta	288.05	2,112.38
F3 to F4 [25]	0.116	0.104–0.129	Beta	270.61	2,062.22
F4 to DC [26]	0.039	0.010–0.079	Beta	3.51	86.48
F4 to HCC [26]	0.014	0.010–0.079	Beta	0.18	12.38
Post F4-SVR to DC [24]	0.008	0.002–0.036	Beta	0.31	38.58
Post F4-SVR to HCC [24]	0.005	0.002–0.013	Beta	1.49	297.13
DC to HCC [27]	0.068	0.030–0.083	Beta	73.58	1008.49
DC (first year) to death from liver disease [27]	0.182	0.065–0.190	Beta	1626.40	7309.88
DC (subsequent year) to death from liver disease [27]	0.112	0.065–0.190	Beta	7.03	55.77
HCC to death from liver disease (26)	0.427	0.330–0.860	Beta	2.14	2.87
***Health state costs (annual in INR)***					
F0-F3 [29, 30]	2000 ($30)^c^	0.5 to 2.0 fold	Gamma	7.11	281.25
Compensated cirrhosis [30]	10,000 ($149)	0.5 to 2.0 fold	Gamma	7.11	281.25
Decompensated Cirrhosis [30]	40,000 ($596)	0.5 to 2.0 fold	Gamma	7.11	1406.25
Hepatocellular Cancer [30]	60,000 ($894)	0.5 to 2.0 fold	Gamma	7.11	5625.00
**Testing cost (INR)**					
Pre-treatment (diagnosis)	8,000 ($119)	0.5 to 2.0 fold	Gamma	7.11	1406.25
Post-treatment	6,000 ($89)	0.5 to 2.0 fold	Gamma	7.11	1125.00
***Health state quality-of-life weights***					
Anemia multiplier ^d^ [31]	0.83	0.75–0.97	Beta	22.95	4.70
F0–F3 [32]	0.93	0.84–0.99	Beta	47.47	3.57
Compensated cirrhosis [32]	0.90	0.81–0.99	Beta	31.12	3.46
DC [32]	0.80	0.57–0.99	Beta	12.29	3.07
HCC [32]	0.79	0.54–0.99	Beta	11.42	3.03
Post-SVR	1.00	0.92–1.00	Beta	3833.92	3.84
**Disutility weights**					
F0-F4 (assumption)	0	0–0.1	Beta	0.15	14.69
DC [33]	0.194	0.127–0.273	Beta	22.58	93.79
HCC [33]	0.508	0.348–0.670	Beta	19.08	18.48

Abbreviations: SVR, sustained virologic response; F0–F4, METAVIR fibrosis score; DC, decompensated cirrhosis; HCC, hepatocellular carcinoma; F4-SVR. Post-SVR state of treated cirrhotic patient. INR, Indian rupees (1 USD = 67.11 INR)

^a^Parameter 1 corresponds to *α* parameter for beta distribution and *k* (shape) parameter for gamma distribution

^b^Parameter 2 corresponds to *β* parameter for beta distribution and *θ* (scale) parameter for gamma distribution

^c^conversion rate:

^d^For patients experienced anemia during treatment, quality of life was multiplied by this factor

### Medical costs

The model represented a healthcare payer’s perspective. The cost of sofosbuvir-ledipasvir (with or without ribavirin), or sofosbuvir-daclatasvir combination treatment was taken as Indian Rupees (INR) 6711 (USD 100) for every 28 days’ supply [6]. The costs of pre-treatment testing (for disease staging and HCV genotyping) was taken as INR 8000 (USD 119), and those for tests while on and after treatment was taken as INR 6000 (USD 89); these costs were estimated using expert opinion, because such data in India are not available (Table 1). We used relatively low costs for treatment of sequelae because many Indian patients, given their limited financial resources, do not access highly-specialized care for these diseases, and because we wanted our model to represent a conservative scenario by underestimating the savings resulting from DAA use; however, this was supplemented by sensitivity analyses using a wide range of costs.

### Quality of life and disability weights

For effectiveness outcomes, we estimated quality-adjusted life years (QALYs), a commonly used metric in cost-effectiveness analysis, and disability-adjusted life years (DALYs), as recommended by the WHO [33]. For QALYs, we assigned quality-of-life (QoL) weights for each health state from previous studies [16, 32, 34]. We assumed the QoL of patients who achieved SVR to be equivalent to that of the general population [32]. However, for those patients with SVR who progressed, the QoL of the respective advanced states was used. To calculate DALYs, we used expected years-of-life lost and disability weights defined by the Global Burden of Disease study (0 for METAVIR scores F0–F4, 0.194 [range 0.127–0.273] for decompensated cirrhosis, and 0.508 [0.348–0.67] for HCC) [33].

### Cost-effectiveness analysis

The MATCH-India model was used to simulate the clinical course of HCV-infected persons in India with and without DAA-based treatment. We ran 10,000 iterations for each profile and projected the expected life-years, discounted QALYs, DALYs and discounted costs. From these, we estimated the incremental cost-effectiveness ratio (ICER; US$ per discounted QALY and US$ per DALY-averted) of DAA-based treatment in comparison with no treatment. We used a lifetime horizon, and discounted both future costs and QALYs at 3% per year, with additional sensitivity analyses with 0% and 5% rates. In addition, we projected the cumulative incidence of decompensated cirrhosis and HCC, and liver-related deaths.

Since HCV progresses slowly, the full benefits of successful HCV treatment may appear several years later. Therefore, we also estimated the ICERs over time. Specifically, we estimated how long would it take for the cost offsets resulting from prevention of advanced disease to exceed the upfront cost of DAA-based treatment.

### Sensitivity analyses

We performed one-way sensitivity analyses to estimate the effects of changes in transition probabilities, QOL weights, disability weights, cost inputs and patient’s age on the cost-effectiveness of treatment. Since our estimates of outcomes of various disease states were derived from developed countries, we conducted a sensitivity analysis considering that mortality rates for various liver conditions in India could be 1.0 to 3.0-fold the baseline rates, and rates of progression of liver fibrosis in HCV infection could be 0.6 to 1.2-fold the baseline. We also performed probabilistic sensitivity analysis using 10,000 first-order and 5000 second-order samples by simultaneously varying all key model inputs using the recommended statistical distributions (Table 1**)**.

## Results

### Baseline cost-effectiveness analysis

Compared with no treatment, the use of DAAs in Indian patients with HCV infection was estimated to increase overall life expectancy by 8.02 years and discounted QALYs by 3.89. The improvement in outcomes was more marked in patients with cirrhosis than those without, and was similar across viral genotypes (Table 2). The no-treatment arm had a lifetime cost of $1,988 per person, entirely for management of advanced sequelae of HCV infection. In contrast, the DAA arm had a lower lifetime cost of $679, including $324 on DAAs, $208 on pre/post-treatment tests, and $147 on management of advanced sequelae that still occurred in a few persons. In other words, antiviral treatment in India was found to be cost-saving, i.e., it increased QALYs by 3.89 years while simultaneously decreasing total healthcare costs by $1,309.

**Table 2:** Cost-effectiveness results comparing model outcomes of no treatment versus treatment with direct-acting antivirals in India.

Patient group	Life Years			Quality-adjusted Life Years (Discounted*)			Total Life-time Cost (Discounted* $)		ICER ($/QALY)**
	No treatment	With DAA-based treatment	Increase in LYs	No treatment	With DAA-based treatment	Increase in QALY	No treatment	With DAA-based treatment	
**Non-cirrhosis (F0–F3)**									
Genotype 1	30.25	37.92	7.677	15.05	18.75	3.71	1,803	536	Cost-saving
Genotype 3	30.25	37.82	7.572	15.05	18.71	3.66	1,803	558	Cost-saving
Genotype 4	30.25	37.70	7.452	15.05	18.65	3.60	1,803	590	Cost-saving
All F0–F3	30.25	37.85	7.600	15.05	18.72	3.67	1,803	553	Cost-saving
**Cirrhosis (F4)**									
Genotype 1	19.16	30.28	11.115	10.37	15.86	5.49	3,182	1,192	Cost-saving
Genotype 3	19.16	29.65	10.487	10.37	15.51	5.15	3,182	1,672	Cost-saving
Genotype 4	19.16	30.51	11.350	10.37	15.97	5.60	3,182	1,152	Cost-saving
All F4	19.16	29.89	10.728	10.37	15.65	5.28	3,182	1,494	Cost-saving
***All patients***	***28*.*76***	***36*.*78***	***8*.*020***	***14*.*42***	***18*.*31***	***3*.*89***	***1*,*988***	***679***	***Cost-saving***

*QALYs were discounted at 3% per year rate

**Cost-saving implies the ICER was negative.

Abbreviations: *DAA*, direct-acting antivirals; *ICER*, incremental cost-effectiveness ratio; *LYs*, life years; *QALY*, quality-adjusted life year

Further, we found that HCV treatment with low-cost DAAs in India would avert 19.07 DALYs per-person treated (Table 3). This gain was similar across HCV genotypes and irrespective of presence/absence of cirrhosis. Cost per DALY averted was negative, i.e., HCV treatment with DAAs was cost-saving.

**Table 3:** Disability-adjusted life years (DALYs) averted with DAA-based treatment in HCV-infected patients in India, in relation to presence or absence of cirrhosis and viral genotype.

Population group	DALY of no treatment	DALY with DAA-based treatment	DALY averted using DAA-based treatment	Cost per DALY averted ($)
**Non-cirrhosis (F0-F3)**				
Genotype 1	19.63	0.35	19.28	-88 (cost-saving)
Genotype 3	19.63	0.58	19.05	-88 (cost-saving)
Genotype 4	19.63	0.94	18.69	-88 (cost-saving)
**All genotypes**	19.63	0.53	19.11	-88 (cost-saving)
**Cirrhosis (F4)**				
Genotype 1	36.36	16.73	19.62	-107 (cost-saving)
Genotype 3	36.36	17.98	18.38	-88 (cost-saving)
Genotype 4	36.36	16.37	19.99	-107 (cost-saving)
**All genotypes**	36.36	17.51	18.85	-95 (cost-saving)
***All patients***	***21*.*87***	***2*.*80***	***19*.*07***	**-89 (cost-saving)**

**Fig 2:**
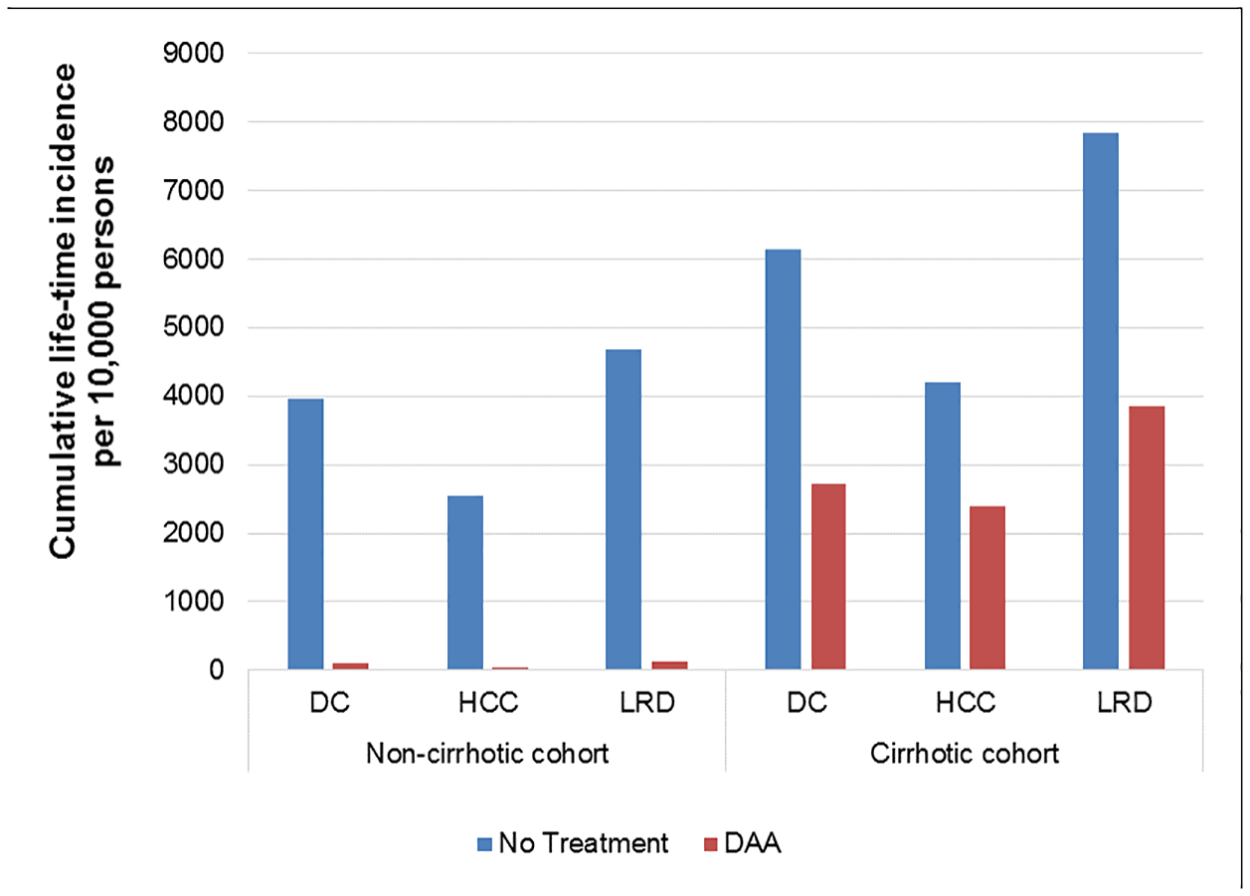
Change in adverse clinical outcomes in patients with hepatitis C virus infection in India following treatment using regimens based on directly-acting antiviral drugs available there at low cost. Abbreviations: DAA, direct-acting antivirals; DC, decompensated cirrhosis; HCC, hepatocellular carcinoma; LRD, liver-related deaths.

Compared with the no-treatment arm, treating 10,000 persons without cirrhosis using DAAs could prevent ~3850 decompensated cirrhosis, ~2500 HCC, and ~4550 liver-related deaths. In cirrhotic patients, treatment could prevent ~3400 decompensated cirrhosis, ~1800 HCC and ~4000 liver-related deaths (Fig 2).

### Cost-effectiveness over time

**Fig 3:**
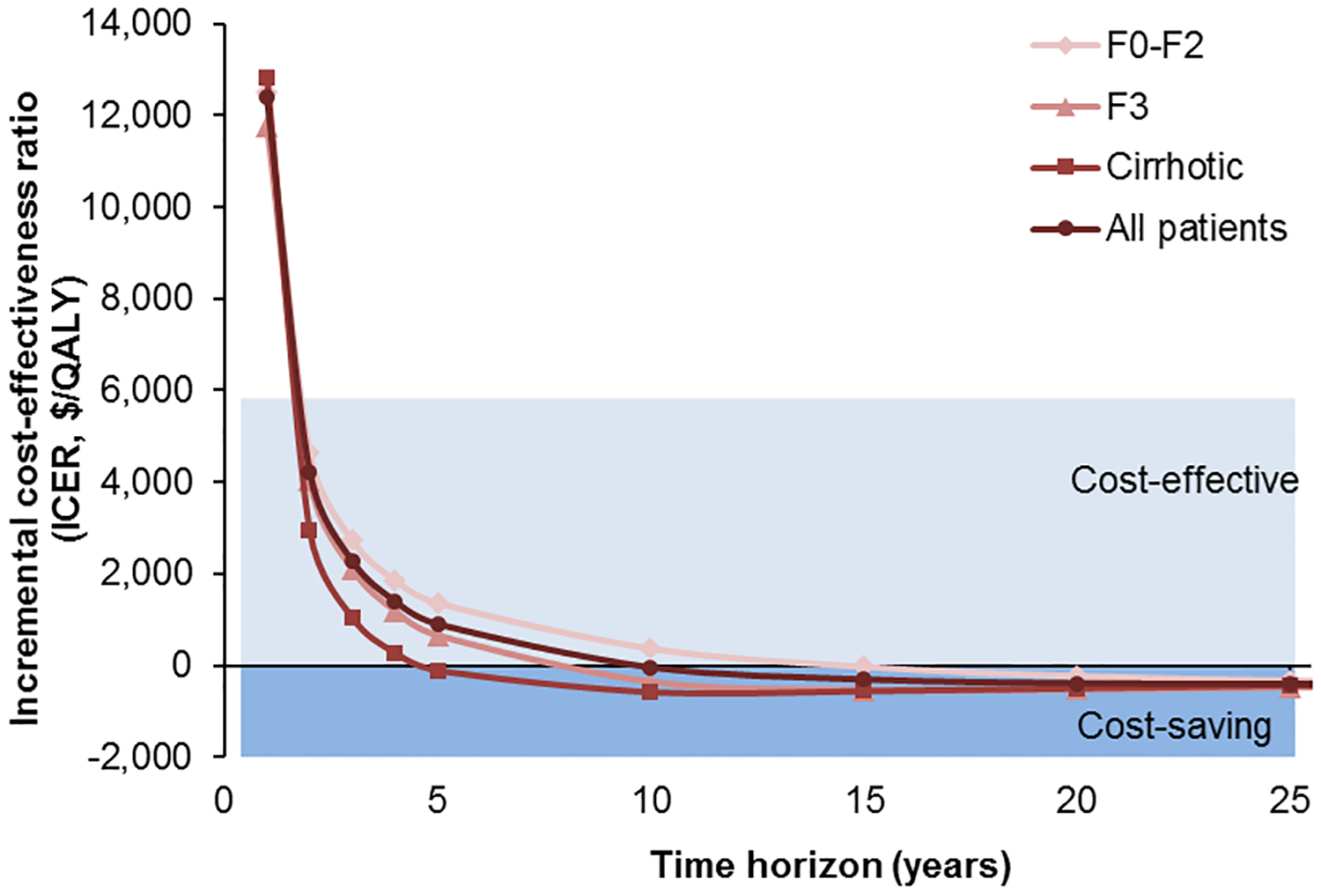
Cost-effectiveness of directly-acting antiviral drug-based treatment of persons with hepatitis C virus infection at various stages of liver fibrosis in India, depending on modelling time horizon.

**Fig 4:**
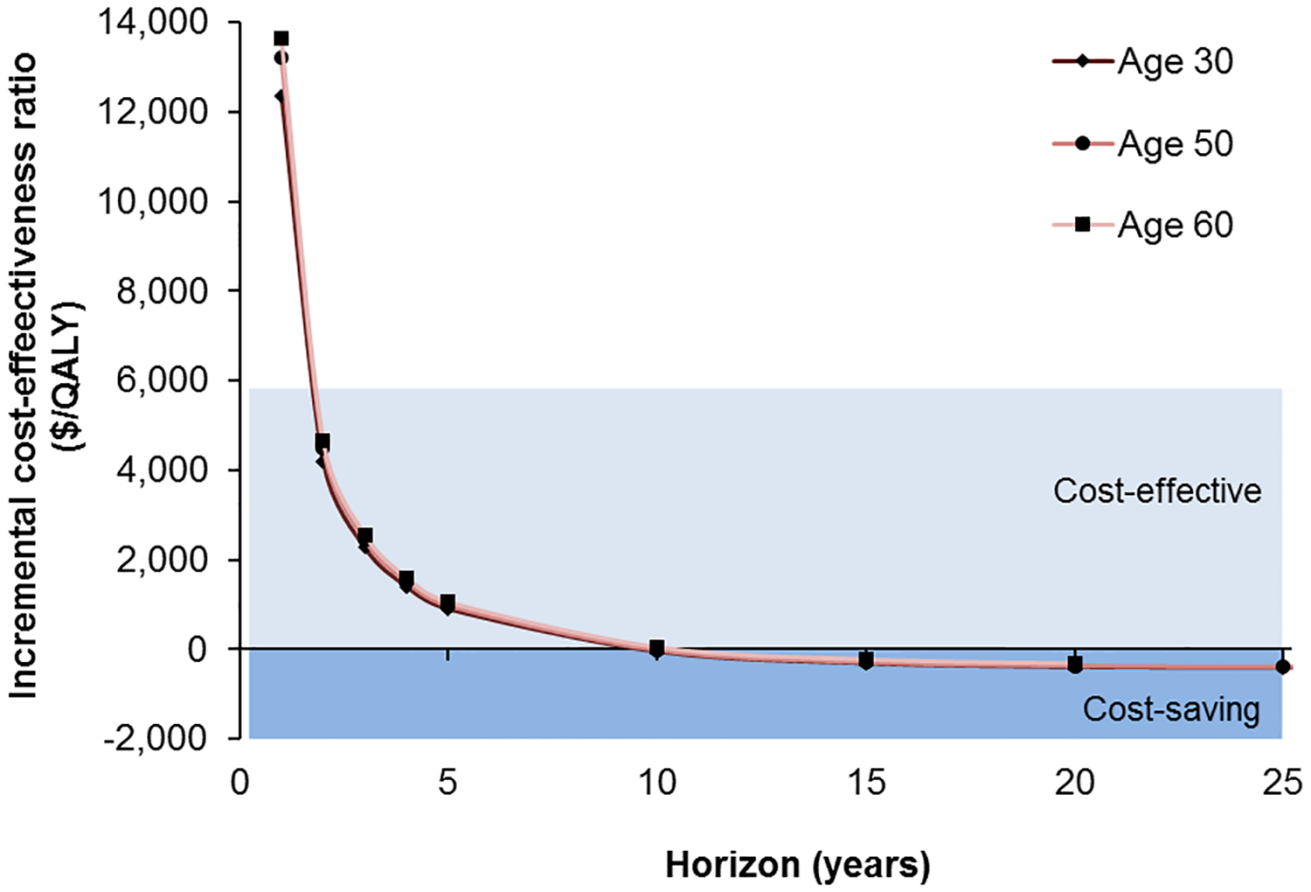
Cost-effectiveness of direct-acting antiviral drug-based treatment of persons with hepatitis C virus infection, by age of starting treatment (years) and time horizon. DAAs became cost-effective within 2 years of initiation of treatment irrespective of patient’s age; and DAAs became cost-saving for patients of age ≤50 around 10 years but never for patients at the age ≥60.

HCV treatment became cost-effective (i.e., ICER < three times India’s per capita annual gross domestic product of $1,942) within 2 years of starting treatment (Fig 3). Furthermore, HCV treatment became cost-saving by 10 years. In other words, by 10 years, the upfront cost of DAAs had been offset by savings from prevention of decompensated cirrhosis and HCC, even if gains in life-years and QALY were ignored. Treatment became cost-saving sooner for patients with advanced disease, i.e. within 4.72 years for those with cirrhosis, and at 11.8 years for those without cirrhosis. We also evaluated the ICER over time for persons treated at different ages (Fig 4); DAAs became *cost-effective* within 2 years and *cost-saving* within 10 years, irrespective of the age at treatment.

### Sensitivity/scenario analyses

**Fig 5:**
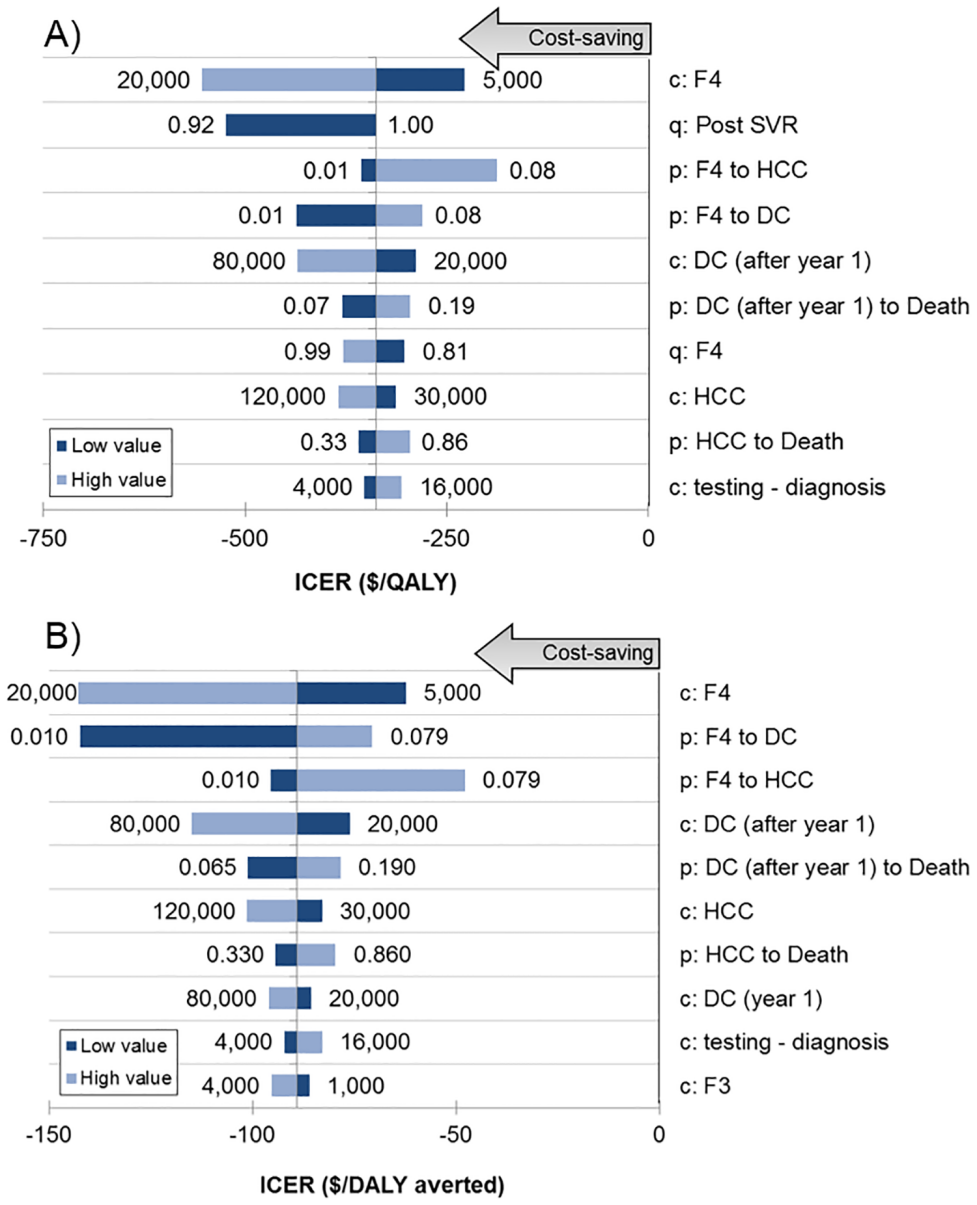
Tornado diagram for one-way sensitivity analysis of incremental cost-effectiveness ratio using (A) $ per additional quality-adjusted life-year, and (B) $ per disability-adjusted life-year averted. Horizontal bars show the variation in incremental cost-effectiveness ratio (ICER; in $/QALY gained or $/DALY averted) with variation in the value of the parameter. In the parameter names, the prefix ‘c’ represents cost of a health-state, ‘q’ the quality-of-life weight and ‘p’ the transition probability from one state to the other. Values of ICER below 0 indicate that the treatment is cost-saving. Abbreviations: QALY = quality-adjusted life-year, DALY = disability-adjusted life-year.

The 10 model parameters to which ICER per QALY was most sensitive are presented in a tornado diagram (Fig 5A). Treatment with DAAs in India always remained cost-saving (ICER/QALY <$0), despite extreme changes in each parameter. The ICER was most sensitive to the costs of management of cirrhosis, post-SVR QoL, and disease progression rate in patients with cirrhosis, cost of HCC management, and cost of HCV diagnosis and testing. Results for ICER per DALY were similar (Fig 5B). In additional scenario analyses on discount rate for costs and QALYs (0% and 5%), age at the time of treatment (20–70 years), drug costs ($100–$900 per 4-week treatment) and mortality rates of liver disease in India compared to developed countries, the DAAs always remained either cost-saving or cost-effective (Table 4). Another scenario analysis showed that DAA treatment remained cost-saving even if rates of progression of fibrosis in HCV infection were different in the Indian population, even though the number of liver events and liver-related deaths did change (Table C in S1 Appendix). A probabilistic sensitivity analysis, which accounted for uncertainty in all model parameters simultaneously, showed DAAs to be cost-saving with a 100% probability (Figure A in S1 Appendix).

**Table 4:** Sensitivity analyses of cost-effectiveness results by age at time of treatment, cost of anti-HCV drug therapy and annual discount rate.

	Cost ($)		QALYs		Incremental QALYs	ICER ($/QALY)
	No treatment	DAA	No treatment	DAA		
**Age category**						
20	2284	700	16.26	21.91	5.65	Cost-saving
25	2207	694	15.78	20.86	5.08	Cost-saving
30	2108	687	15.14	19.64	4.49	Cost-saving
35 (base case)	1988	679	14.42	18.31	3.89	Cost-saving
40	1847	669	13.54	16.83	3.29	Cost-saving
45	1683	658	12.60	15.36	2.75	Cost-saving
50	1495	646	11.47	13.73	2.27	Cost-saving
55	1291	632	10.27	12.09	1.83	Cost-saving
60	1090	617	8.94	10.34	1.40	Cost-saving
65	893	599	7.61	8.64	1.03	Cost-saving
70	711	586	6.27	7.04	0.77	Cost-saving
**Drug cost (4 weeks)**						
$100 (base case)	1988	679	14.42	18.31	3.89	Cost-saving
$300	1988	1325	14.42	18.31	3.89	Cost-saving
$600	1988	2295	14.42	18.31	3.89	79
$900	1988	3265	14.42	18.31	3.89	329
**0% discount rate**						
Non-cirrhosis (F0–F3)	3387	597	23.61	31.94	8.33	Cost-saving
Cirrhosis (F4)	4714	2052	14.66	25.04	10.38	Cost-saving
All patients (F0–F4)	3565	792	22.41	31.02	8.61	Cost-saving
**5% discount rate**						
Non-cirrhosis (F0–F3)	1275	537	11.86	14.20	2.34	Cost-saving
Cirrhosis (F4)	2572	1293	8.61	12.25	3.64	Cost-saving
All patients (F0–F4)	1449	639	11.42	13.94	2.52	Cost-saving
**Hazard ratio for liver-related mortality in India compared to our baseline estimate**						
1.00	1988	679	14.42	18.31	3.89	Cost-saving
1.25	1868	663	14.31	18.29	3.99	Cost-saving
1.50	1778	652	14.22	18.28	4.07	Cost-saving
1.75	1713	644	14.15	18.28	4.12	Cost-saving
2.00	1657	638	14.09	18.27	4.17	Cost-saving
2.50	1577	627	14.01	18.26	4.25	Cost-saving
3.00	1519	620	13.95	18.25	4.30	Cost-saving

## Discussion

Hepatitis C is a major cause of morbidity globally, with estimated 495,000 deaths and loss of 12.6 million DALYs per year [3]. The availability of DAAs has however changed the HCV treatment paradigm, leading to hope of elimination of this infection as a public health threat by the year 2030 [35]. However, the affordability and cost-effectiveness of DAAs, particularly in low-resource settings, have been debated widely. In our analysis, we found that HCV treatment with low-cost DAAs available in India will result in cost-savings because of prevention of decompensated cirrhosis and HCC in future. Furthermore, upfront spending on DAAs will provide a good value for money within as soon as 2 years of initiation of treatment.

Previous studies on cost-effectiveness of DAA-based treatments for HCV have all focused on the situations in developed countries, such as United States and European Union, where the drug costs are high [36]. In these studies, such treatment was found to be cost-effective, i.e. had ICER per QALY of below annual per capita national income, even at high costs prevalent in those countries. However, despite this demonstrated cost-effectiveness, the uptake of HCV treatment in these settings has been lower than expected because of the high cost of treating all infected patients.

By contrast, in several low-income countries, DAAs are available at highly discounted prices. Our cost-effectiveness analysis showed that the treatment of HCV infection with DAAs at the prices prevalent in India was not only cost-effective, but was in fact cost-saving, i.e., the cost of treatment is offset by the savings in future healthcare costs, thus leading to an overall monetary saving while at the same time increasing QALYs. Situations such as this, where pharmacological treatments have been found to be cost-saving, are rare. Thus, HCV treatment with low-priced DAAs is a win-win situation, warranting the use of public money to fund it.

Our results provide a compelling case for India to invest in HCV treatment. Unlike most other pharmacological interventions, HCV treatment with generic DAAs is not merely cost-effective, but also cost-saving. Therefore, investment in HCV treatment paying back for itself over time could be a very rewarding public health investment. For that reason, we estimated the duration in which the upfront cost of DAAs will be offset by the resultant health benefits—a useful parameter for policymakers who prioritize allocation of funds across sectors and diseases. Our analysis found this ‘pay-back’ time to be as short as 10 years, making the public funding of DAA-based treatment of HCV in India quite attractive. In this context, it seems particularly worthwhile to fund the treatment of patients with compensated cirrhosis, since the expenditure would be recouped even faster, i.e. within 5 years.

We recognize that our study had some limitations. First, we did not include patients with HCV genotypes 2, 5 and 6 because their prevalence rate is less than 1% in India. Second, as with most of the previously published models of HCV treatment, we ignored the benefits and costs of re-treatment of patients who failed DAAs. With recent advancements in DAAs, newer compounds that are effective in patients who had not responded to the drugs considered in our analysis. However, these newer drugs, which should be useful for retreatment, are not yet available in India; also, these drugs too are expected to be available at low prices in India. Third, the costs of treatment of HCV sequelae in our study were based on expert opinion than on real data, because of lack of published information. However, our conservative assumptions, if anything, biased the analysis against the DAA-based treatment; further, sensitivity analyses over a wide range of costs showed that our results were robust. Finally, we excluded patients with HIV or hepatitis B virus co-infection, or those at higher risk of HCV reinfection. However, this should not detract from our conclusions since such patients account for only a small proportion of the total HCV-infected pool in the Indian population. On the positive side, our analysis and report follow the principles recommended by a WHO group, aimed at enhancing transparency of economic analyses in the field of viral hepatitis [17]. Of course, such analyses would gain from better cost data on various disease conditions in different settings, an area that needs further work.

Health planners would be interested in knowing whether the treatment is more cost-effective or cost-saving in some particular subgroups, which could then receive priority for treatment. We found that gender and viral genotype did not influence the cost-effectiveness of DAA-based HCV treatment much. However, patient’s disease stage and age at treatment influenced the response, with a much greater cost-effectiveness in those with advanced liver disease. Hence, the governments with limited resources may consider prioritizing treatment of such groups, to maximize the public health impact within their limited resources [37]. Such prioritization is often essential, since ramp-up of public-health treatment programs takes time, and the entire budget is seldom immediately available. Another factor that may guide prioritization is the regional variation in HCV epidemiology within a country; in such cases, modelling for various subnational regions may be helpful.

Though the cost of DAAs has come down, the cost of diagnosis and testing remain high. Thus, with the availability of pan-genotypic DAA-based regimens, it would be important for public-health programs to develop and implement simplified treatment guidelines that obviate the use of pre-treatment HCV genotype testing. Similarly, it would be useful to limit the post-treatment testing to only one occasion, i.e. 12 weeks after completing treatment [37]. Also, in future, use of HCV core antigen test as a cheaper alternative to HCV RNA testing, and reducing the cost of testing through negotiations with service providers, could make HCV treatment with DAAs even more economically attractive. Though our study looked only at persons already diagnosed to have HCV infection, bringing down the testing cost could also help make population-level HCV screening and treatment programs more cost-effective.

The licensing agreements that have made low-cost generic DAAs available in India, allow for sale of these drugs in several other low- and low-middle-income countries at low prices. Hence, our results should generally apply to these other countries too, both for individual case treatment and for public-funded treatment programs.

In conclusion, we found that low-cost generic DAAs available in India can improve patient outcomes and also result in cost-savings in a fairly short time-frame. Therefore, HCV treatment should be a priority for health planners of this country, and other countries where low-cost DAAs are available, not only from the public health viewpoint, but also from the economic perspective.

## Supporting information

S1 Appendix (DOCX)
