# Supplementary material for: Cost-effectiveness of hepatitis C treatment using generic direct-acting antivirals available in India
Source: PLoS One. 2017 May 17;12(5):e0176503. doi: 10.1371/journal.pone.0176503 (PMC5435174; doi:10.1371/journal.pone.0176503)
Supplement: S1 Appendix — (DOCX) [file pone.0176503.s001.docx]

**SUPPLEMENTARY APPENDIX**

**Cost-Effectiveness of Hepatitis C Treatment using Generic Direct-Acting Antivirals Available in India**

Rakesh Aggarwal, MD, DM,^1^ Qiushi Chen, PhD,^2,3^ Amit Goel, MD,DM,^1^ Nicole Seguy, MD, MPH,^4^ Razia Pendse, MD, MPH,^5^ Turgay Ayer, PhD^6^ Jagpreet Chhatwal, PhD,^2,3^

^1^Department of Gastroenterology, Sanjay Gandhi Postgraduate Institute of Medical Sciences, Lucknow, India

^2^Massachusetts General Hospital Institute for Technology Assessment, Boston, MA USA

^3^Harvard Medical School, Boston, MA USA

^4^World Health Organization India Country Office, New Delhi, India*

^5^World Health Organization Regional Office for the South-East Asia, Communicable Diseases Department, New Delhi, India*

^6^H. Milton Stewart School of Industrial and Systems Engineering, Georgia Institute of Technology, Atlanta, GA USA

*****The author is a staff member of the World Health Organization. The author alone is responsible for the views expressed in this publication and they do not necessarily represent the decisions or policies of the World Health Organization.

**Table A. Assumptions used for baseline population distribution of various characteristics among HCV-infected persons in India**

| Parameter | Value |
| --- | --- |
| Age [1] | 35 (20–70) |
| Fibrosis score [2] |  |
| F0 | 18.4% |
| F1 | 24.8% |
| F2 | 21.7% |
| F3 | 21.7% |
| F4 | 13.4% |
| Sex[1] |  |
| Male | 58% |
| Female | 42% |
| Virus genotype* [3] |  |
| G1 | 32.0% |
| G3 | 63.4% |
| G4 | 4.6% |

Abbreviations: HCV, hepatitis C virus; F, METAVIR fibrosis score; G, genotype

Data for age are shown using base case assumption and range

*HCV genotypes 2, 5 and 6 were not considered because of their rarity in India.

**Table B. Treatment regimens used based on HCV genotype and patients’ liver fibrosis stage, and the corresponding expected rates of sustained virological response (SVR), treatment discontinuation and adverse events (AEs)**

| HCV genotype | METAVIR fibrosis stage | Treatment drugs | Treatment duration (weeks) | SVR (%) | Treatment discontinuation rate (%) | AEs  (anemia) (%) | Duration of AEs (weeks) |
| --- | --- | --- | --- | --- | --- | --- | --- |
| G1[4] | F0-F3 | SOF + LDV | 12 | 98.1 | 1 | 1 | 2 |
|  | F4 | SOF + LDV | 12 | 93.2 | 1 | 1 | 2 |
| G3[5, 6] | F0-F3 | SOF + DCV | 12 | 97.0 | 0 | 1 | 4 |
|  | F4 | SOF + DCV | 24 | 86.0 | 2 | 1 | 4 |
| G4[7] | F0-F4 | SOF + LDV | 12 | 95.0 | 1 | 2 | 2 |

Abbreviations: DCV = daclatasvir, LDV = ledipasvir

**Table C. Sensitivity analysis of rates of progression of HCV-related liver disease on cost-effectiveness of HCV treatment and various liver-related events and mortality**

|  | HR=0.6* | HR=0.8* | HR=1 (base) | HR=1.2* |
| --- | --- | --- | --- | --- |
| ICER ($/QALY) | -318  (cost-saving) | -331  (cost-saving) | -337  (cost-saving) | -339  (cost-saving) |
| Number of years needed to be |  |  |  |  |
| Cost-effective | 1.8 | 1.8 | 1.8 | 1.8 |
| Cost-saving | 11.1 | 10.1 | 9.8 | 9.5 |
| Cumulative liver-related outcomes per 10,000 persons |  |  |  |  |
| Non-cirrhotic |  |  |  |  |
| DC: No Treatment | 2822 | 3475 | 3959 | 4379 |
| DC: DAA | 79 | 98 | 112 | 124 |
| DC: Prevented | 2744 | 3377 | 3847 | 4255 |
| HCC: No Treatment | 1799 | 2238 | 2547 | 2815 |
| HCC: DAA | 29 | 29 | 29 | 29 |
| HCC Prevented | 1770 | 2209 | 2518 | 2786 |
| LRD: No Treatment | 3277 | 4078 | 4674 | 5191 |
| LRD: DAA | 92 | 113 | 128 | 142 |
| LRD: Prevented | 3186 | 3965 | 4546 | 5048 |
| Cirrhotic |  |  |  |  |
| DC: No Treatment | 6149 | 6149 | 6149 | 6149 |
| DC: DAA | 2729 | 2729 | 2729 | 2729 |
| DC: Prevented | 3420 | 3420 | 3420 | 3420 |
| HCC: No Treatment | 4211 | 4211 | 4211 | 4211 |
| HCC: DAA | 2383 | 2383 | 2383 | 2383 |
| HCC Prevented | 1828 | 1828 | 1828 | 1828 |
| LRD: No Treatment | 7846 | 7846 | 7846 | 7846 |
| LRD: DAA | 3859 | 3859 | 3859 | 3859 |
| LRD: Prevented | 3987 | 3987 | 3987 | 3987 |

*Abbreviations*: HR= hazard ratio, ICER = incremental cost-effectiveness ratio, QALY = quality-adjusted life years, DAA = direct-acting antivirals, DC = decompensated cirrhosis, HCC = hepatocellular carcinoma, LRD = liver-related deaths.

*We applied hazard ratios to change the probability of transition from F0 to F1, F1 to F2, F2 to F3 and F3 to F4 using the formula: $p^{'}=1-\left( 1-p \right)^{HR}$


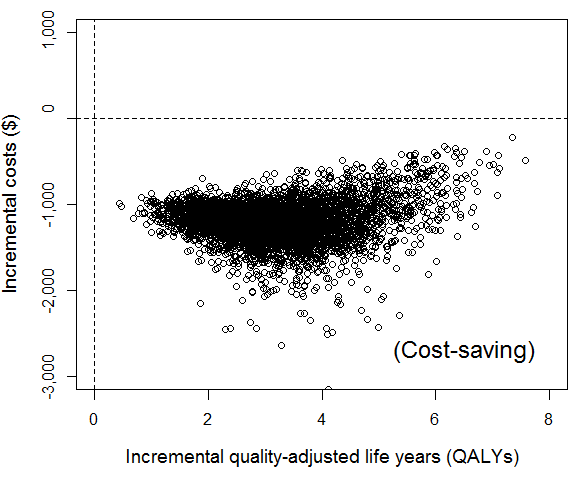


**(a)**

**
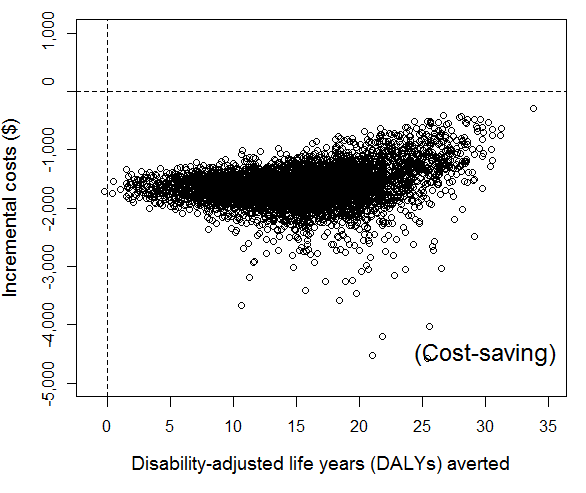
**

**(b)**

**Figure A. Scatter plot of incremental cost and effectiveness (QALYs) for probabilistic sensitivity analysis. (a) Incremental cost vs. incremental QALYs, (b) incremental cost vs. DALY-averted.** The figure shows 5,000 Monte Carlo runs of comparison of outcomes. Each of the 5000 runs showed that DAAs would improve QALYs, avert DALYs and reduce total healthcare costs.

**References**

1. Chowdhury A, Santra A, Chaudhuri S, Dhali GK, Chaudhuri S, Maity SG, et al. Hepatitis C virus infection in the general population: a community-based study in West Bengal, India. Hepatology (Baltimore, Md). 2003;37(4):802-9. Epub 2003/04/02. doi: 10.1053/jhep.2003.50157. PubMed PMID: 12668973.

2. Ahmad W, Ijaz B, Javed FT, Gull S, Kausar H, Sarwar MT, et al. A comparison of four fibrosis indexes in chronic HCV: development of new fibrosis-cirrhosis index (FCI). BMC gastroenterology. 2011;11:44. Epub 2011/04/22. doi: 10.1186/1471-230x-11-44. PubMed PMID: 21507271; PubMed Central PMCID: PMCPMC3098184.

3. Narahari S, Juwle A, Basak S, Saranath D. Prevalence and geographic distribution of Hepatitis C Virus genotypes in Indian patient cohort. Infection, genetics and evolution : journal of molecular epidemiology and evolutionary genetics in infectious diseases. 2009;9(4):643-5. Epub 2009/05/23. doi: 10.1016/j.meegid.2009.04.001. PubMed PMID: 19460332.

4. Afdhal N, Zeuzem S, Kwo P, Chojkier M, Gitlin N, Puoti M, et al. Ledipasvir and sofosbuvir for untreated HCV genotype 1 infection. The New England journal of medicine. 2014;370(20):1889-98. Epub 2014/04/15. doi: 10.1056/NEJMoa1402454. PubMed PMID: 24725239.

5. Nelson DR, Cooper JN, Lalezari JP, Lawitz E, Pockros PJ, Gitlin N, et al. All-oral 12-week treatment with daclatasvir plus sofosbuvir in patients with hepatitis C virus genotype 3 infection: ALLY-3 phase III study. Hepatology (Baltimore, Md). 2015;61(4):1127-35. doi: 10.1002/hep.27726. PubMed PMID: 25614962; PubMed Central PMCID: PMCPMC4409820.

6. Welzel TM, Petersen J, Herzer K, Ferenci P, Gschwantler M, Wedemeyer H, et al. Daclatasvir plus sofosbuvir, with or without ribavirin, achieved high sustained virological response rates in patients with HCV infection and advanced liver disease in a real-world cohort. Gut. 2016. doi: 10.1136/gutjnl-2016-312444.

7. Kohli A, Kapoor R, Sims Z, Nelson A, Sidharthan S, Lam B, et al. Ledipasvir and sofosbuvir for hepatitis C genotype 4: a proof-of-concept, single-centre, open-label phase 2a cohort study. Lancet Infect Dis. 2015;15(9):1049-54. doi: 10.1016/S1473-3099(15)00157-7. PubMed PMID: 26187031; PubMed Central PMCID: PMCPMC4561573.
